# Α Quantum Pattern Recognition Method for Improving Pairwise Sequence Alignment

**DOI:** 10.1038/s41598-019-43697-3

**Published:** 2019-05-10

**Authors:** Konstantinos Prousalis, Nikos Konofaos

**Affiliations:** Department of Informatics, Aristotle University of Thessaloniki, Thessaloniki, Greece

**Keywords:** Genome assembly algorithms, Machine learning, Protein sequence analyses, Software, Quantum information

## Abstract

Quantum pattern recognition techniques have recently raised attention as potential candidates in analyzing vast amount of data. The necessity to obtain faster ways to process data is imperative where data generation is rapid. The ever-growing size of sequence databases caused by the development of high throughput sequencing is unprecedented. Current alignment methods have blossomed overnight but there is still the need for more efficient methods that preserve accuracy in high levels. In this work, a complex method is proposed to treat the alignment problem better than its classical counterparts by means of quantum computation. The basic principal of the standard dot-plot method is combined with a quantum algorithm, giving insight into the effect of quantum pattern recognition on pairwise alignment. The central feature of quantum algorithmic -quantum parallelism- and the diffraction patterns of x-rays are synthesized to provide a clever array indexing structure on the growing sequence databases. A completely different approach is considered in contrast to contemporary conventional aligners and a variety of competitive classical counterparts are classified and organized in order to compare with the quantum setting. The proposed method seems to exhibit high alignment quality and prevail among the others in terms of time and space complexity.

## Introduction

Figuring out similar portions among two or more strings of symbols is a complicated computing task as different degrees of similarity may be considered. Determining the correspondences between a target subsequence and a reference sequence consists a hard but also a qualifiedly tractable problem known as sequence alignment (SA) or mapping. The alignment of sequences remains an active research area of great importance for natural language processing, business and marketing research, forensics, ecology, epidemiology, medicine, and especially in biological studies. Mapping and studding segments of various genomes has become indispensable for basic research in the interdisciplinary field of genomics. The alignment process rearranges sequences introducing spaces, or gaps, making insertions or deletions (indels), translocation and inversion operations, in order to achieve the optimal matching. Mutation or other evolutionary natural mechanisms are usually the reasons for all these random modifications on the original biological sequences.

Modern sequencing technologies are described by the term ¨Next Generation Sequencing¨ (NGS)^1^, a.k.a. parallel or deep sequencing. These technologies achieve ultra-high throughput sequencing allowing billions of bases being sequenced within a single day, and more specifically they produce the *reads* which are smaller portions of a genome sequence of a living organism. Genome databases are continuously loaded with experimentally generated raw sequence data resulting in huge amounts of unprocessed clusters of reads.

Separately from the remarkable progress of sequencing technologies, current computational methods of structural and functional determination of biological sequences create an increasing gap, due to their slower and costly implementation. The alignment of sequences can generally be performed in two ways: (a) the pairwise sequence alignment and (b) the multiple sequence alignment. The first way considers only two sequences at a time whereas the second considers more than two related sequences at a time. Obviously, multiple alignment is more advantageous than pairwise since it provides more information. However, the implementation of multiple alignments is a difficult task as it seeks solutions to more complex methods such as heuristics or repetitive optimization strategies. In any case, multiple alignment approaches are highly based on pairwise alignment methods.

The first approach to align the similarity between two sequences is traced back to 1970, when Gibbs and McIntyre^2^ introduced the similarity matrix, also known as dot-plot. Due to its limited capacity to encompass and analyze large sequences, other methods were inquired in ¨dynamic programming¨ (D.P.). In particular, the inventions of Smith-Waterman^3,4^ (S.W.) and Needleman-Wunsch^5^ (N.W.) algorithms were the greatest examples in D.P. These algorithms are based on a scoring framework providing optimal alignment solutions. The performance of both S.M. and N.W. algorithms depends on the lengths of the reference *S*_*R*_ and the query *S*_*Q*_ sequences, *L*_*R*_ and *L*_*Q*_ respectively, achieving *O*(*L*_*R*_*L*_*Q*_) runtime and memory resources. Then, the word methods (a.k.a. *k*-mer or *k*-tuple) were introduced to speed-up the alignment process. In the last two decades, combinatorial strategies were invented like seed-and-extend and q-gram filter to optimize the speed and the accuracy of the alignment result.

On the parallel, the field of pattern recognition has been extended over the quantum information and computing settings. In 2001, both R. Schützhold and C.A.Trugenberger set the concept of quantum pattern recognition (QPR). R. Schützhold mentions a quantum algorithm which runs on a probe structure line those in diffraction experiments and detects easily patterns^6^. Trugenberger proposed the quantum analogue of the classical associative memory^7,8^ confronting the problem of capacity shortages but probabilistically retrieving incomplete or noisy inputs. R. Zhou and Q. Ding mention in 2008 an alternative way^9^ of QPR with 100% probability to retrieve the query pattern which is highly based on an improved version of Grover’s search algorithm. Using quantum multi-pattern search algorithm^10^ may be possible to achieve pairwise SA but such a technique demands a relatively complex way in producing quantum original states if a high number of patterns exist. In 2009, R. Neigovzen *et al*. describe a notable QPR scheme^11^ which according to its authors combines the idea of a classical Hopfield neural network with adiabatic quantum computation and E. A. Fard *et al*. present a quantum neural network^12^.

In this study, we expand our previously reported method^13^ which seems to outperform its classical counterparts at least in terms of time and computing resources. The reported method explains how it is possible to turn R. Schützhold’s pattern recognition schema into a novel alignment approximation by utilizing the central concept of the old but infallible technique of dot-matrix plots. In fact, the crossing points of the dots on the matrix are simulated onto an array structure lattice plane like those in diffraction experiments offering a different approach to the alignment process.

It is assumed that our aligner is a complex device which can handle large sequences on a lattice plane surface made by non-linear Kerr media and run R. Schützhold’s QPR algorithm in a quantum computing system. The physical realization of such a device may be an apparatus consisting of a Laue diffraction system with a probing light beam (e.g. X-rays or electrons) and two computational subunits: one with a classical processor and one with a quantum processor. The R. Schützhold’s QPR algorithm is analytically described in the Methods section splitting it into five steps. The most complex parts are the quantum black box^14^, or *BB*, and the quantum Fourier transform (QFT)^14,15^. The classical computing subunit is needed to run calculations of Laue’s equations in order to localize and specify candidate line-patterns.

## Specification of the QPR Circuit

**Figure 1:**
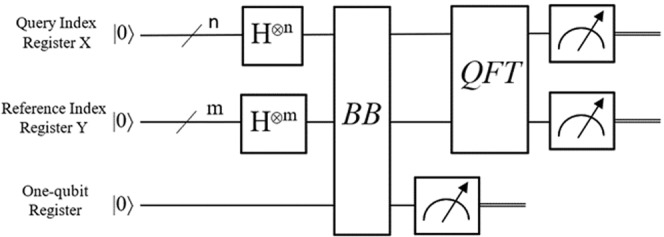
Circuit model of R. Schützhold’s quantum pattern recognition method.

According to R. Schützhold’s QPR algorithm two registers are used as an index structure for the formation of a dot-plot like diffraction pattern. The *regX* register for the horizontal and the *regY* register for the vertical indexing. A third one-qubit register is entangled with the other two registers and a single measurement of it let in superposition only the dot cells (reflective cells). Then the QFT enhances the amplitude of the superposition at the proper index coordinates on the plane revealing the existence of prominent line patterns. A few more runs of the algorithms may be needed to secure the detection result and some further classical calculations will locate the pattern. The circuit model of the quantum part of this algorithm is depicted in Fig. 1.

In an over-simplifying way, the action of a *Hadamard* transform (gate *H*) on |0〉 in Fig. 1 sets the two coordinate registers into a superposition of the input state. Assuming that the X and Y coordinate registers have *n* and *m* qubits respectively, the expression ${H}^{\otimes n}$ or ${H}^{\otimes m}$ denotes this action. The operator of the *H* gate is defined in Eq. (1).

1 $$H\equiv \frac{1}{\sqrt{2}}[\begin{array}{cc}1 & 1\\ 1 & -1\end{array}]$$

The symbol “⊗” represents the tensor product. The coordinate registers come into a superposition by the application of the Hadamard gates which is described by Eq. (2).

2 $$|regX,Y\rangle \equiv \frac{1}{\sqrt{{2}^{n+m}}}\sum _{x=0}^{{2}^{n+m}-1}|x\rangle $$

The *BB* sub-circuit acts similarly to the black boxes of the milestone algorithms of P. Shor and L. Grover. The application of a unitary operation as *BB* affects the state of each qubit concurrently and this is *quantum parallelism*. Actually, this can be viewed as a function *f*(*x*) that evaluates for many different values of *x* simultaneously, as *f*(*x*): {0, 1}^*L*^ → {0, 1}. Starting from the state |*ψ*_0_〉 = |*x*〉|0〉 then we see that *BB*|*ψ*_0_〉 = |*x*〉|*f*(*x*)⊕0〉 = |*x*〉|*f*(*x*)〉. Since the domain is a binary *w*-bit string and the range a one-bit, it is possible to consider *x* as the address of a cell in the rectangular array and the one-bit result *f*(*x*) as a flag identifier on whether the cell is a reflective one or not. The unitary operation of the *BB* may be constructed by phase shift gates *R*_*u*_ of $|\pi |,|\frac{\pi }{2}|,|\frac{\pi }{{2}^{2}}|\ldots ,or\,|\frac{\pi }{{2}^{n+m-1}}|$ turns as the next unitary operation is the *QFT*. The *R*_*u*_ unitary transformation, a.k.a. *phase-Shift*, is defined in Eq. (3),

3 $${R}_{u}\equiv [\begin{array}{cc}1 & 0\\ 0 & {e}^{2\pi i/{2}^{u}}\end{array}]$$

and its operation modifies the phase *φ* = 2*π*/2 ^*u*^ (where $u\in {\mathbb{Z}}$) of a hypothetical quantum state $|q\rangle =\alpha |0\rangle +\beta {e}^{-i\phi }|1\rangle$ leaving unchanged the probability of measuring the orthonormal basis states of the quantum system. Moreover, the *BB* includes the application of *controlled NOT* or *cNOT* gates which are necessary to develop entanglement between the coordinate registers and the one-qubit register allowing for a number of parallel computation paths. The *cNOT* gate is defined by the operator in Eq. (4).

4 $$cNOT\equiv [\begin{array}{cc}\begin{array}{cc}1 & 0\\ 0 & 1\end{array} & \begin{array}{cc}0 & 0\\ 0 & 0\end{array}\\ \begin{array}{cc}0 & 0\\ 0 & 0\end{array} & \begin{array}{cc}0 & 1\\ 1 & 0\end{array}\end{array}]$$

Right after the measurement, the system |*ψ*〉 remains in a superposition as in Eq. (5) and entangled, but only for the states that either had *f*(*x*) = 0 or *f*(*x*) = 1.

5 $$|\psi \rangle \equiv \frac{1}{\sqrt{{2}^{n+m}}}\sum _{x=0}^{{2}^{n+m}-1}|x\rangle |f(x)\rangle $$

The next sub-circuit is the *QFT* (see the circuit in Supplementary Material). It is the well-known linear transformation that affects the amplitudes and phases of the base states of a qubit-system. Τhe first set of the input qubit states |*q*_1_〉..|*q*_*m*_〉, where *m* < *u*, represents the qubits of the *regY* input register and the rest |*q*_*m+1*_〉..|*q*_*u*_〉 the qubits of the *regX* input register. Typically, the number of gates that are required to build *QFT* circuit grows only as a quadratic function of the number of the input qubits. From bottom up, *QFT* circuit has 1 + 2 + … + *u* = *u*(*u* + 1)/2 elementary *Hadamard* and controlled rotation gates. Such an implementation has an exponential speed up over the classical fast Fourier transform. Moreover, the approximate version of *QFT* known as *AQFT* can improve further the performance but this study is out of our scope.

### Proposed SA protocol

**Figure 2:**
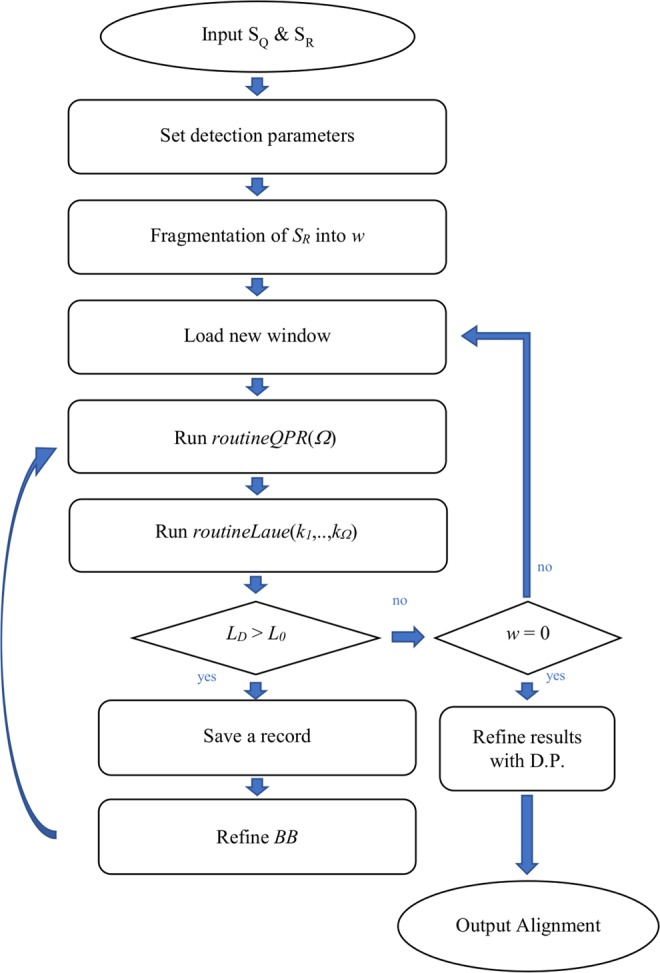
Pairwise alignment protocol’s flow-chart based on the heuristic method of quantum pattern recognition. Routine routineQPR is repeated *Ω* times for each window space w and routineLaue is supplied with *Ω* measurement values *k*_*i*_. A window is exhausted when the detected line has length *L*_*D*_ less than a threshold *L*_*0*_.

The protocol of our method is described in Fig. 2. In the same way to the dot matrix comparison, the user has to input the query *S*_*Q*_ and the reference *S*_*R*_ sequences and investigate the plane for diagonal line patterns. Reference’s length *L*_*R*_ may be a relatively small number when a local alignment is conducted or a large one when a global alignment or database searching takes place. If the size of *S*_*R*_ is large enough, a fragmentation process splits it into smaller segments for a more efficient management. These segments are also known as window spaces. Then the *routineQPR* quantum routine runs *Ω* times per window to find and secure the largest line pattern in the region under comparison and then *routineLaue* classical routine locates the position of this pattern within this window space. *routineLaue* also estimates the length of the line pattern *L*_*D*_ and the deviation angle -π/2 ≤ *ϑ* ≤ π/2 from a vertical one. A record with all the relevant information about the detected line is saved and the procedure continues till all the windows are exhausted. Given a line pattern starting at *x*_0_ and *y*_0_ on the panel should keep the following information: *record* (*id*, *L*_*D*_, *ϑ*, [*x*_0_, *x*_0_ + *L*_*D*_], [*y*_0_, *y*_0_ + *L*_*D*_]). Finally, a list of records is formed for each segment and algorithms like S.W., N.W., FOGSAA, or modified versions of them undertake for the optimal alignment.

Dot matrix analysis provides a great way to compare every position in the matrix. Its usefulness is seen in alignment of proteins, nucleic acid sequences, finding amino acid repeats within a protein (comparing it by itself), database searching or sequence assembly.

The formed diagonals in the dot matrix plane, illustrate the degree of similarity. All the occurring diagonal lines that are formed right after *BB*’s application may not be of the same importance since most of them may emerge from the small alphabet of the sequences which causes a lot of noise (e.g. nucleic acids in DNA has a value of *α* = 4). Diagonals may not be perfectly shaped up to the degree that the density of dots *ρ* within a line-width (e.g. *L*_*D*_/2) deviates by an acceptable quantity *Δρ* from the mean *ρ*. A threshold value (or cutoff value) for the minimum acceptable length *L*_0_ of detected diagonals should be defined in the beginning of the protocol in order to trim redundant or insignificant matches. But as it is the case in most situations, more than one diagonal line will occur in a typical window space with a small alphabet. Multiple occurring lines in a confined region may be a confusing problem for our probabilistic QPR algorithm in two directions:

- When line patterns of nearby lengths exist in the same window, it will be difficult to distinguish them in their superposition by *QFT*, as they form coherently amplitudes of the same measuring probability. The longer the length of a diagonal line in contrast to the other diagonals, the easier the way to detect it. However, this case is usually limited in microsatellite repeats or patterns.
- When two, or more, line patterns of accepted lengths, >*L*_0_, exist in the same window, then the lengthiest of them will always shade the others rendering difficult their detection.

One way to combat the first problem is to adjust properly the size *L*_*W*_ of the window. *L*_0_ should always be a few orders of magnitude smaller than *L*_*W*_. Reducing *L*_*W*_ the probability to allow more than one acceptable diagonal in it shrinks. Another way to consider is to include classical heuristic methods, but increasing the computational load.

An option to treat the second problematic area is to invent a way to destroy the amplitudes of the first detected diagonal within a single window space. It can be possible by adding some extra phase gates in the *BB* to direct properly the quantum Fourier transformation. Another easier and cost-effective option would be to remove the region of the previously detected diagonal in *S*_*R*_, but this solution would omit to take into consideration smaller and acceptable diagonals that occur within the range of the previously detected diagonal. However, for small *L*_*W*_ this option may be an effective solution. A third option would be to include again classical methods.

*L*_0_ and *L*_*W*_ are two critical parameters which have to be carefully chosen with the aid of statistics. The goal of analysis usually determines the window size *L*_*W*_ and the threshold length *L*_0_ of diagonal. The size of the average exon or the average protein structural element, of gene promoter or enzyme active site, are typical factors that affect window’s size. The level of noise in a dot matrix for a small *α* may cause the occurrence of irrelevant diagonals but since the significant diagonals have a good similarity and aggregate many dots then the detection effectiveness is independent of noise. Thus, a densely dot matrix with a high *ρ* will demand a higher threshold length at the risk to encompass irrelevant diagonals. A small window size would increase dramatically the number of the windows and therefore the application times of *routineQPR*. An interesting improvement would be to examine first the diagonal windows for a large-scale alignment.

The size of the diagonal will affect the number of measurements that we have to apply. The larger the length, the higher the probability to be measured, as small patterns need more measurements^6^ to secure the result. If the size of a line pattern is a small portion *β* of the array size *L*_*Q*_*L*_*R*_, the probability to locate a line pattern is given by Eq. (6).

6 $$p=O(\frac{\beta {\rm{\Delta }}{\rho }^{2}}{\rho })$$

The repetition number *Ω* of *routineQPR* has an upper limit *O* $(\frac{1}{\sqrt{{\beta }_{min}}})$ in respect to the size of the accepted line-pattern.

## Results

The aim of this work is to examine whether the QPR method can be used efficiently as an alternative to the conventional sequence alignment methods. The performance of our QPR protocol is tested in terms of computational resources and run time. In order to evaluate its contribution to the alignment procedure, a simple program was implemented to simulate the behavior of the novel QPR algorithm (see Methods section). Though the adopted algorithm isn’t far from reality, quantum computing technology isn’t yet mature enough to carry out efficiently the subroutines *QFT* or *BB* for multiple qubits (>20). Quantum entanglement fragility and decoherence render unstable quantum computation. The performance of our method is tested and some pairwise alignment examples are presented to demonstrate the precision of our method as a benchmark test with known aligners. For the following results, the supportive data will be found in the Supplementary Material.

### Computing resources

The circuit in Fig. 1 provides a clear and compact summary of the quantum computing resources that are needed for this method. The two memory registers *regX* and *regY* and a set of single-qubit quantum gates including: *Hadamard*, *cNot* and *phase-Shift* operations, are the fundamental components for the implementation of the proposed *routineQPR* routine.

**Figure 3:**
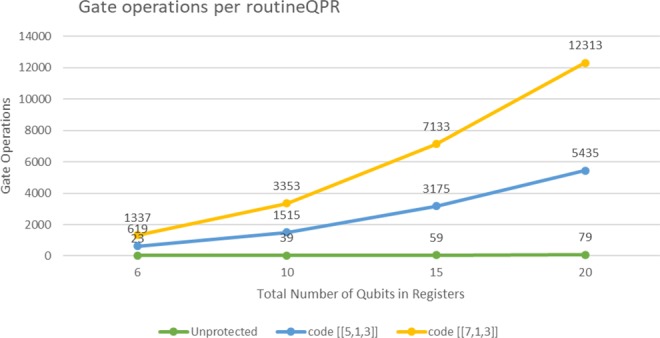
Gate operations per routineQPR execution. regX and regY ’s qubits are considered as one unified memory space.

In order to conduct a theoretical analysis with numerical treatments, an optimistic specification of the *routineQPR* ’s hardware is assumed which is based on two realistic implementations on different technology platforms. The first one is the superconducting transmon IBM-QE device with limited qubit connectivity, and the other is a fully connected trapped-ion system^16^. Both are limited to 5-qubits, but we do the assumption that we can reach the size of 20 in each register as it is enough to index sequences of more than one million base pairs (bp). According to their specifications the execution of an *X*, *Hadamard* and *cNOT* gate takes 130 ns, 130 ns, and 650 ns for the superconductor and approximately 20000 ns, 20000 ns, and 250000 ns for the ion-trap system, assuming the aforementioned memory size. For a more thorough estimation, the encoded versions of the circuit are compared with the unencoded. The well-studied distance-3 [[5,1,3]] and [[7,1,3]] error correcting codes are included in the estimation chart in Fig. 3.

An increment in the qubits of the registers, has a sublinear augmentation on gate operations. Quantum recovery protocols usually add an expensive computing overhead. Error correction takes place after one or two logical gates. Generally, gate operations of the encoding and recovery system occupy more than 90% of the overall system. In this circuit the approximated version of QFT is considered with a degree of 2.

From the aspect of memory allocation, our proposed method needs only a few qubits. Quantum registers can host sequences of size that increases exponentially in the number of qubits. Only a little classical memory will be needed to keep records of the matching points.

### Run time estimation

Given the hardware specification and the QPR algorithm, the expected run time of the routineQPR routine is estimated by dividing the algorithm mainly into three computing steps: the initial superposition by *Hadamards*, the *BB* application and the *QFT* subroutine. The run time escalation for 6, 10, 15 and 20 totally indexing qubits is described in Table 1. *Hadamard* and *phase-Shift* gates are supposed that act concurrently and parallel. Noise recovery delay is not included.

**Table 1:** Runt time estimation per routineQPR execution.

	Time in μs			
Total register’s size (L_Q_ + L_R_) in Qubits	**6**	**10**	**15**	**20**
Superconducting Transmon Qubits	2,29	3,81	5,71	7,61
Trapped Ions	1410	2490	3840	5190

**Figure 4:**
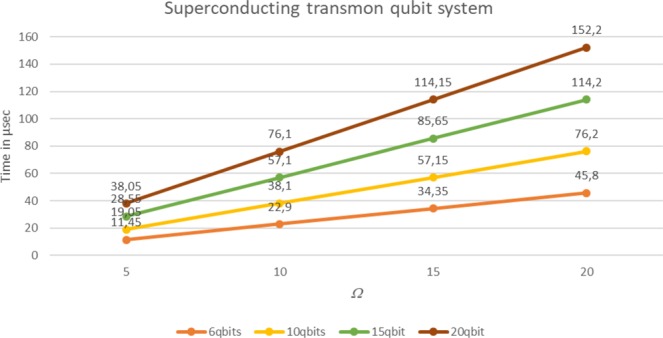
Time consumption of parameter *Ω*.

As parameter *Ω* secures pattern recognition and detection, its behavior for more memory qubits is studied. This parameter is critical for the performance of our method, as it may be time consuming for many repetitions of *routineQPR(Ω*). In the following chart time consumption is calculated for 6, 10, 15 and 20 qubit memory in the range 5 ≤ *Ω* ≤ 20. The results in Fig. 4 demonstrate that the size of the register affects *Ω* significantly, thus a good management of this parameter will save much time. However, run times still remain at relatively low levels.

### SNP calling accuracy

Some model species of the bacteria *Escherichia sp*. are used in this section to compare the SNP-calling performance of our simulation with that in a recent research^17^ by four well-established alignment tools CUSHAW (v.1.0), SOAP2 (v.2.2.1), Bowtie (v.0.12.7) and BWA (v.0.7.0). In particular, a pair of alignment is used between the *E*. *coli K12 MG1655* (accession number *SRR*001*665* in NCBI SRA) and the *E*. *coli 536* (accession number *NC_008253* in GenBank) using the paired-end alignment for each aligner. According to this work, 20.8 million 200-bp-insert-size paired-end reads of the *SRR00*1*665* are aligned onto the reference genome *NC_008253*. The first three columns in Table 1 provide the precision and recall results for CUSHAW, SOAP2 and BWA.

Due to the large size of the sequences our classical simulator took many hours to approximate the alignment quality levels of the classical aligners. Real reads of medium size 200 bp were extracted from the query sequence *SRR001665* in order to treat mapping as it would be in a single read sequencing way and not as in paired-end. A sample of ½ million reads covers almost more than ¼ of the initial sample which is a secure amount to approximate and consider our comparison. Then, the reduced reference genome is divided into segments of size 1000 bp and the window size is defined at read’s length. Each read is tested over the reference genome and when an acceptable line occurs, the threshold *L*_*0*_ criterion is fulfilled and the window is recorded as “positive” in order to keep track of the alignment. The threshold value *L*_*0*_ has an elasticity of 15–20% mismatches in each window. Each time that a positive window occurs, it is recorded as a positive hit by the simulator. Due to some shifted diagonals the width of the examination of the neighboring windows was increased in order to cover more thoroughly problematic regions with highly distorted diagonals. The output results are finally compared with the peer reviewed alignments as they are reported in the relative study^17^.

The SNP calling is evaluated by using the precision and recall measures. Precision is defined as TP/(TP + FP) and recall as TP/(TP + FN). TP stands for true positive and FP stands for false positive, representing a match and mismatch, respectively, with a SNP (Single Nucleotide Polymorphism). FN is a false negative and represents a “correct” SNP that was not identified.

The final comparison results between the four popular aligners and our own method is presented in Table 2. The QPR method seems to be advantageous as the precision and recall measures surpass the other methods having a relatively small standard deviation. By a short research, the average time of a single mapping of a read for the four aligners was estimated, in order to conduct the runtime comparison. Moreover, the runtimes for the single-end (SE) and the paired-end (PE) alignment methods are included as derived by the aforementioned research^17^ to demonstrate the superiority of our method. The parameter *Ω* has a sufficient number of times to detect more than one diagonal lines in a single window.

**Table 2:** Benchmarking alignment test between classical aligners and QPR method (*Ω* = 6) querying *E. coli* K12 MG1655(SRR001665) on *E. coli* 536. The SD measure is included in QPR.

	CUSHAW	SOAP2	Bowtie	BWA	QPR Method (by simulation)
Precision	90,00%	90,84%	93,47%	91,82%	94,24% (±1.48)
Recall	97,51%	92,85%	84,75%	97,22%	97,83% (±0.85)
**Average time per read mapping (in** ***ms*** **)**					
SE	1,154	2,044	1,343	3,721	0,00642
PE	2,898	2,310	3,013	3,929	
**Approximated runtime (in** ***s*** **)**					
SE	1026	2617	1085	4764	28,84
PE	3711	2958	3858	5031	

### Computational complexity

To investigate in a uniform way all the established known short read alignment methods, five stages were considered to cover every stage of these strategies. In the first stage all the preliminary operations take place regarding the reference system. In the second and the third stage the generation and the mapping process are examined, respectively. The fourth and the fifth stages deal with the mapping validity of the candidate matching list. Twelve popular aligners plus our own method and its classical counterpart are analyzed to demonstrate the pros and cons of each strategy in Table 3.

**Table 3:** Comparison of Sequence Alignment Strategies in terms of Computational Complexity.

PSA Tool	Year	Strategy	Stage 1	Time & Space Complexity	Stage 2	Time & Space Complexity	Stage 3	Time & Space Complexity	Stage 4	Time & Space Complexity	Stage 5	Time & Space Complexity
Title Name		General method	Construction of the Indexing Structure		Seed Generation/Organization		Seed Searching (or seeding)		Mapping & Extension		D.P. Alignment	
GNUMap	2009	*Seed & Extend k*-mer exact	Hash *S*_*R*_	*T*: *O* (*L*_*R*_ + (*L*_*R*_ log *L*_*R*_)) *S*: *O* (*L*_*R*_)	PWM + quality filter	*T*: *Ο* (*L*_*PWM*_*L*_*R*_)	Hash table	*Avg*: *Θ* (1) *Worst*: *O* (*L*_*S*_)	Quality Filter	*T*: *Ο* (*N*_*m*_)	Probabilistic NW scoring	*T*: *Ο* (*L*) *S*: *Ο* (*L*)
SOAP2	2009	*Seed & Extend k*-mer inexact	Suffix trie BWT-FM *S*_*R*_	*T: Ο* (*L*_*R*_ + (*L*_*R*_ log *L*_*R*_)) *S*: *O* (*L*_*R*_)	Read split	—	Suffix trie	*Avg*: *Θ* (1) *Worst*: *O* (*L*_*S*_)	—	—	S.W. scoring	*T*: *Ο* (*L*) *S*: *Ο* (*L*)
Bowtie	2009	*Seed & Extend k*-mer inexact	Suffix trie Bidirectional BWT-FM *S*_*R*_	*T: Ο*(*L*_*R*_ + (*L*_*R*_log*L*_*R*_)) *S: O*(*L*_*R*_)	Read split	—	Suffix trie	*Avg*: *Θ* (1) *Worst*: *O*(*L*_*S*_)	Base-calling quality score	*Avg*: *Θ* (1) *Worst*: *O*(*L*_*S*_) *Space*:*Ο* (2 *L*_*S*_)	S.W. scoring	*T*: *Ο* (*L*) *S*: *Ο* (*L*)
BWA	2009	*Seed & Extend k*-mer inexact	Prefix trie *S*_*Q*_	*T: Ο*(*L*_*R*_) *S*: *O*(*L*_*R*_)	Prefix DAWG *S*_*s*_	*T*: *Ο* (*L*_*S*_) *S: O* (*L*_*S*_)	Prefix trie	*Avg*:*Θ*(1) *Worst*: *O*(*L*_*S*_)	Overlapping criterion	*O*(*N*_*m*_)	S.W. scoring	*T*: *Ο* (*L*) *S*: *Ο* (*L*)
RMAP	2008	*Seed & Extend k*-mer spaced	—	—	Hash seed table Base-calling filtration	*T*: *O* (*r*) + *O* (log *L*_*S*_) *S*: $O({a}^{\frac{{L}_{S}}{k}}+rk)$	Hash table	*O*(*L*_*G*_)	Base-calling quality score	*Avg*: *Θ* (1) *Worst*: *O*(*L*_*S*_) *Space*:*Ο* (2 *L*_*S*_)	S.W. scoring	*T*: *Ο* (*L*) *S*: *Ο* (*L*)
Maq	2008	*Seed & Extend k*-mer spaced	Hash *S*_*R*_	*T*: *O* (*L*_*Q*_ + (*L*_*Q*_ log *L*_*Q*_)) *S*: *O* (*L*_*Q*_)	Seed list exact & inexact	*T*: *Ο* (*L*_*R*_) *S: O* (*L*_*R*_)	Hash table	*Avg*: *Θ*(1) *Worst*: *O*(*L*_*S*_)	Base-calling quality score	*Avg*: *Θ* (1) *Worst*: *O*(*L*_*S*_) *Space*:*Ο* (2 *L*_*S*_)	S.W. scoring	*T*: *Ο* (*L*) *S*: *Ο* (*L*)
BWA-MEM	2013	*Seed & Extend* MEMs	Bidirectional FMD-index *S*_*Q*_	*T: Ο* (*L*_*Q*_ + (*L*_*Q*_ log *L*_*Q*_)) *S*: *O* (*L*_*Q*_)	Two rounds of SMEMs	*T*: *Ο* (*L*_*Q*_ + *L*_*R*_ + *k* + *1*) *S: Ο* (*L*_*S*_)	FMD-index	*Worst*: O(*L*_*S*_)	Ranking cutoff	*~O*(*N*_*m*_)	S.W. affine gap penalty	*T*: *Ο* (*L*) *S*: *Ο* (*L*)
CUSHAW2	2014	*Seed & Extend* MEMs	FM-ind. suffix trie for *S*_*G*_ or *S*_*Q*_	*T: Ο*(*L*_*Q*_ + (*L*_*Q*_ log *L*_*Q*_)) *S*: *O*(*L*_*Q*_)	Finding MEMs	*T*:*Ο* (*L*_*Q*_ + *L*_*R*_) *S: Ο* (*L*_*S*_)	Suffix tree FM-index	*Ο* (*L*_*S*_ + *L*_*G*_)	—	—	S.W. scoring	*T*: *Ο* (*L*) *S*: *Ο* (*L*)
LAST	2011	*Seed & Extend* Adaptive	Suffix tree *S*_*R*_	*T: Θ* (*L*_*R*_) *S: Θ* (*L*_*R*_)	Spaced suffix array of adaptive seeds	*T:Ο* (*L*_*S*_) *S: Ο* (*L*_*S*_)	Suffix tree	*Ο* (log *L*_*S*_)	—	—	S.W. scoring	*T*: *Ο* (*L*) *S*: *Ο* (*L*)
AMAS	2015	*Seed & Extend* Adaptive	Suffix tree *S*_*R*_	*T: Θ* (*L*_*R*_) *S: Θ* (*L*_*R*_)	Spaced suffix array of adaptive seeds	*T:Ο* (*L*_*S*_) *S: Ο* (*L*_*S*_)	Suffix tree N_m_ < Threshold	*Ο*(log *L*_*S*_)	Already 95% candidates out	*~O*(*N*_*m*_)	S.W. scoring	*T*: *Ο* (*L*) *S*: *Ο* (*L*)
SHRiMP	2011	Spaced q-grams	Hash table *S*_*R*_	*T*: *O*(*L*_*R*_ + (*L*_*R*_ log*L*_*R*_)) *S*: *O*(*L*_*R*_)	q-gram index construction	*T*: *O*(*L*_*Q*_ + *L*_*S*_ *L*_*R*_ *a*^*-q*^) *S*: *O*(*L*_*Q*_ + 4^*q*^)	Hash table	*Avg*: *Θ*(1) *Worst*: *O*(*L*_*S*_)	—	—	Vectorized S.W. scoring	*T*: *Ο* (*L*) *S*: *Ο* (*L*)
Hobbes	2012	q-grams	Hash table *S*_*R*_	*T*: *O*(*L*_*R*_ + (*L*_*R*_ log *L*_*R*_)) *S*: *O*(*L*_*R*_)	q-gram index construction	*T*: *O*(*L*_*Q*_ + *L*_*S*_ *L*_*R*_ *a*^*-q*^) *S*: *O*(*L*_*Q*_ + 4^*q*^)	Hash table	*Avg*: *Θ*(1) *Worst*: *O*(*L*_*S*_)	Apply filtering	*~O*(*N*_*m*_)	S.W. or N.W. scoring	*T*: *Ο* (*L*) *S*: *Ο* (*L*)
—	—	**Proposed**	**Diffraction pattern (BB** + **Meas/ment)**	***T: O*** **(log**_**2**_ ***L*** **)** ***S*** **:** ***Ο*** **(log**_**2**_ ***(L***_***R + ***_ ***L***_***Q***_ **))**	—	—	**QFT**	***O*** **(log**_**2**_ ***L*** **)**	—	—	**D**.**P**. **scoring or not**	*T*: *Ο* (*L*) *S*: *Ο* (*L*)
Classical Analogue	—	Dot-matrix	Table construction for *S*_*R*_ and *S*_*Q*_	*T*: *O* (*L*_*R*_ + *L*_*Q*_) *S*: *O* ((*α* + 4) *L*_*R*_ + *2L*_*S*_)	—	—	FFT		$(lo{g}_{2}^{q+1}L)$	—	—	D.P. scoring or not

Notes:*S*_*Q*_ and *L*_*Q*_ are the query read sequence and its length.

*S*_*S*_ and *L*_*S*_ are the seed sequence and its length.

*S*_*R*_ and *L*_*R*_ is the reference genome sequence and its length.

*q* is the length of a substring (*q*-gam) of a target sequence.

*L* = *L*_*S*_*L*_*Q*_,

*L*_*PWM*_ is the length of a PWM sequence.

*N*_*m*_ is the matching times,

*r* is the number of the seed strings in a seed-table,

*k* is the allowed mismatches in the search,

*a* is the number of all the different elements of the sequence (*a* = 4 for DNA seq. and *a* = 20 for protein seq.).

*The outcome of the quantum measurements should be extracted immediately.

In order to allow for a vivid comparison between our method and the various alignment tools, Table 3 summarizes all the ingredient algorithms of modern aligners with their time and space complexities. This comparative elaboration demonstrates the intrinsic computational power of each aligner. Apart from the old dot-matrix method^18^, most of the other aligners are some prominent variations^19,20^ of the seed-and-extend strategy: (1) the *k*-mer exact match seed (BLAST^21^ and GNUMap^22^), (2) the *k*-mer inexactly match seed (SOAP^23,24^, Bowtie^25,26^, BWA^27^), (3) the *k*-mer spaced seed (RMAP^28^, Maq^29^), (4) the maximum extend match^30^ (MEM) seed (BWA-MEM^20^, CUSHAW2^31^) and (5) the adaptive seed (LAST^32^, AMAS^33^). For the *q*-gram filter strategy, the SHRiMP^34^ and Hobbes^35^ are examples with improved sensitivity.

The fundamental tools that manage the sequences in all these aligners are: the hash map, the prefix/suffix trie^36^, the Burrows-Wheeler Transform with FM-index^37,38^, the FMD-index^39^, the bidirectional BWT^40^ or the Directed Acyclic Word Graph^41^. Further information about them can be found in the Supplementary Material.

The comparison table shows that the quantum approach outperforms the other classical strategies in terms of computational complexity as at every stage the quantum setting has lower complexity than its classical counterparts. The usage of hash tables or suffix trees impose the preparation of indexing structure which may take time and space. Moreover, their manipulation may not be affordable enough in certain cases. Hash tables turn out to be more efficient than search trees or any other lookup table structure, but hash collisions are practically unavoidable when hashing a random subset of a large set of possible keys. A data structure based on Burrows-Wheeler Transform can search stored items in constant time which is the best possible complexity, but in reality, the complexity may range between *Θ* (1) and *O*(*L*_*Seed*_) where *L*_*Seed*_ the length of the query seed. The QPR approach is evolved in a dynamic way binding only a few memory qubits. The quantum index structure can host exponentially larger data segments of genome sequences in the increase of the number of qubits.

As the goal of Table 3 is to describe the computing complexities, it is not clear what is the real consumption of resources. The computational overhead to conduct seed generation is unnecessary in the QPR approach since the *QFT* can detect the line-patterns in a logarithmic time. The repetitive seed generation stages, the various calculations for seed or mapping scoring and filtering and the final alignment processes based on D.P. imply a poorer performance, even if stage 2 provides an advantage with the constant time complexity by the searching methods. However, the S.W. or N.W. or alternative of dynamic programming should be incorporated in our method in order to achieve precision similar to the other classical aligners.

For weak similarities or particular patterns, the QPR approach may fail to detect or interpret correctly positive windows and further study may improve the situation. Obviously, reducing window’s size or increasing *Ω* surely will increase sensitivity at a cost of time reaching a level that will limit the need for dynamic programming. Long reads are the future tendency in NGS techniques and longer inexact or spaced seeds can guarantee an accurate matching detection. In this regime, the quantum algorithm may have a great advantage as it supports the detection of arbitrary long imperfect line-patterns.

## Discussion

A sophisticated method is presented which combines dot-matrix plotting and quantum pattern recognition to improve sequence alignment in biology research and all the relevant fields. The results of our experiments lead to the conclusion that there is a great advantage with a method which should be explored further in the future. The implementation of such a method is not far from the reality, since it does not demand a coherent quantum system of many qubits.

The overall time complexity of our method is *O*(3log_2_*L*_*R*_*L*_*Q*_). The first term concerns the application of the Hadamard gates, the second the application of the *BB* and the third the QFT. The final aligning step is common for all the strategies, but it may be limited in dot-matrix approaches. Current aligners seem that they cannot run faster than *O*(*L*_*R*_*L*_*Q*_) in the worst case. Moreover, the quantum registers can save much space due to the exponential indexing coverage of the sequences.

Since, the QPR approach isn’t directly an alignment algorithm, the combination with classical tools may seem unavoidable but better treatments can be employed to make perfect alignments. Using scoring methods will also improve the alignment result. More benchmarking tests are needed under specific circumstances to illustrate the power of this method. Database searching and sequence assembly may be benefited. Another area to be consider with the QPR method is the multiple sequence alignment.

## Methods

### RoutineQPR

The primary task of this method is to detect, identify and localize certain linear patterns in an unstructured data set as it is argued by R. Schützhold in his seminal paper^6^. The considered linear pattern in this study is the line pattern. The first part of this method uses quantum computation while the second part uses classical computation.

A full unstructured data set has to be loaded into a lattice plane which can be considered as a rectangular *n* × *m* array. This array is assumed to consist of unit cells (e.g. controlled refractors or any non-linear Kerr cells) which are, either completely absorptive, or completely reflective. The reflective cells are also assumed to be uniformly distributed in the rectangular array with a density *ρ* < 1. The position of the reflective points on the plane may be located by shining appropriately focused light beams on the Kerr media and doing calculations in close analogy to diffraction experiments studying crystal structures via Laue diagrams. Symmetrical pattern transformations are assumed to be resilient for an ideal number of times into different directions.

In another way, the array data can be treated by a quantum black box (*BB*) of the form in Eq. (7). The *BB* transformation is based on the principal of quantum parallelism and acts as a binomial classifier and selector for the array data. The input *x* and *y* quantum states, being in a superposition, can encode simultaneously all the coordinates of the reflective cells in the array. The initial *x* and *y* quantum states are superpositions that represent all the possible coordinates as *n*- and *m*- qubit strings laying in quantum registers. The zero-state register consists of a single qubit which has to come into entanglement with the other two registers. A single measurement of the entangled qubit will randomly determine which of the groups of the binomial classification will survive, which means that then the *x* and *y* states will exclusively become superpositions, either of the reflective, or the absorptive, cells.

7 $$BB:[\begin{array}{c}|x\rangle \\ |y\rangle \\ |0\rangle \end{array}]\to [\begin{array}{c}|x\rangle \\ |y\rangle \\ |f(x,y)\rangle \end{array}]$$

Since the unstructured data set is loaded into the array structure, the method is ready to begin. The whole method can be translated into five algorithmic phases:

#### Input data preparation

The two coordinate registers are initialized into |0〉 state and then the Hadamard transformation is applied. This step is described in Eq. (8). *N* and *M* parameters represent the rows and the columns of the rectangular array, respectively.

#### Black-Box run

The BB transformation is applied and the third one-qubit registered is measured. The current state of the registers is described by Eq. (8). If the outcome of the measurement is 1, the resulting superposition concerns only the reflective cells. Otherwise, the resulting superposition concerns only the absorptive cells (the complementary set) and step 2 is repeated.

8 $$BB[\begin{array}{c}{H}^{(n)}|{0}^{(n)}\rangle \\ {H}^{(m)}|{0}^{(m)}\rangle \\ |0\rangle \end{array}]=\frac{1}{\sqrt{NM}}\sum _{x=0}^{N-1}\sum _{y=0}^{M-1}[\begin{array}{c}|x\rangle \\ |y\rangle \\ |f(x,y)\rangle \end{array}]$$

After a successful measurement, Eq. (8) will obtain the form of Eq. (9). The *NM* product in Eq. (9) is reduced to *ρNM* or *ρS*, considering that *S* = *NM*. Coordinate registers *x* and *y* can be considered as a single one under the symbol *z*, |*z*〉 = |*x*〉 ⊗ |*y*〉. The complex number *z* (*z* = *x* + *Ny*) can be viewed as an *s*-digit binary number which indicates a point in the array in the form of a string made by the coordinates *n* + *m* = *s*. The current superposition is given by the quantum state |*Ψ*〉 with 0 ≤ ${z}_{\ell }$ ≤ *S* − 1.

9 $$|{\rm{\Psi }}\rangle =\frac{1}{\sqrt{\rho S}}\sum _{\ell =1}^{\rho S}|{z}_{\ell }\rangle $$

#### QFT application

It is time to apply the QFT circuit network to the |*z*〉 superposition state. The final result is described in Eq. (10).

10 $$QFT|{\rm{\Psi }}\rangle =\sum _{k=0}^{S-1}\sum _{\ell =1}^{\rho S}\frac{1}{S\sqrt{\rho }}\exp \,(2\pi i\frac{{z}_{\ell }k}{S})|k\rangle $$

At this stage, some useful information about the pattern can be extracted by measuring |*k*〉. Some values of *k* may be more prominent as their amplitudes may be increased by the QFT. Line patterns demonstrate a stable repetition of reflective cells developing peaks of |*z*〉 factor. These values of *k* are known as wave-numbers and can lead to the detection of the pattern (a.k.a. feature selection). When there isn’t a prominent pattern then the measurement of the quantum state *k* yield just noise.

#### Pattern localization

This is the only classical part of this method since it makes use of classical computation. By using the equations of Laue diagrams used in diffraction experiments it is possible to find the location of the detected patterns and extract further information about their length and their direction. This step demands a second execution of the algorithm.

#### Fidelity verification

A few more repetitions of the algorithm may be needed to ensure the detection of our findings.

### RoutineLaue

In a continuation of the QPR algorithm, the key parameters *L*_*D*_ and *ϑ* can be inferred from the measurements in *routineQPR*, in order to locate the center points *z* of a particular diagonal which is described by

11 $$z={z}_{{0}}+{[{\bf{N}}(N+\tan \vartheta )]}_{integer}$$

where **N** is an integer. The *Ω* measurements [*k*_*1*_, *…*, *k*_*Ω*_] of *routineQPR* running for a single window are stored and used in this routine. Some peaks are generated in each row of the pattern

12 $$k={[{\bf{N}}\cos \vartheta \frac{S}{{L}_{D}}\pm {{\rm O}}(\frac{M}{{L}_{D}\sqrt{\chi }})]}_{integer}$$

while the summation of the peaks of all rows interferes constructively only if *k* is fine-tuned according to

13 $$k={[{\bf{N}}\cos \vartheta \frac{N-\tan \vartheta }{N}\pm {\rm O}(\frac{1}{\sqrt{\chi }})]}_{{\rm{integer}}}$$

The second term in Eqs (12) and (13) denotes the width of the peak. The associated Laue function *f*_*Laue*_(*ξ*,*κ*) = sin2(πξκ)/sin2(πκ), with *k* = *κ*cos *ϑ* *S*/*L*_*D*_ and *ξ* = *O*(*N* $\sqrt{\beta }$) for the case of Eq. (12) and *κ* = *k*(N + tan*ϑ*)/S and *ξ* = *O*(M $\sqrt{\beta }$)) for the case of Eq. (13) can lead to the calculation of the position and the width of the peaks.

Prevalent peaks in the measurements of *k* occur for values which satisfy both conditions in Eqs (12) and (13), concurrently. So, the wave-numbers of the potential peaks are given by

14 $$k\approx [{\bf{N}}\,\cos \,\vartheta \frac{S}{{L}_{D}}\pm \,\sin \,\vartheta \frac{M}{{L}_{D}}]$$

However, Eq. (14) cannot give large peaks standalone. The first few of them may be suppressed, but some potential peaks from Eq. (14) may match conditions (12) and (13). One more time, the same process has to be repeated for the transposed array (*NM*→)*MN*. The weave-numbers of the peaks are now

15 $$k^{\prime} \approx {[{\bf{N}}\sin \vartheta \frac{S}{{L}_{D}}\pm \cos \vartheta \frac{N}{{L}_{D}}]}_{{\rm{integer}}}$$

since transposing changes the array orientation per *π*/2.

So far, the candidate values *L*_*D*_ and *ϑ* can be approximated. Using the candidate values for *L*_*D*_/cos *ϑ* from Eq. (14) and *L*_*D*_/sin *ϑ* from Eq. (15) a mathematical system is created for solution. Therefore, comparing the condition Eqs (12) and (13) and the sets of the suppressed peaks will provide accurately the *L*_*D*_ and *ϑ* values.

For the size of the pattern, the frequency of measuring the peaks at *k* and *k΄* and their width is enough to find fraction *β*. So, with the aid of *L*, *ϑ* and *β*, the pattern can be localized efficiently by splitting up symmetrically the window into smaller orthogonal windows and running *routineQPR* again in these smaller windows. A richer analysis of this method can be found in the seminal paper^6^.

### Classical simulation of *routineQPR*

A simple program is implemented by using the bioinformatics toolbox application of Mathworks in the Matlab suite.

The readily available function of *seqdotplot* allows the creation of a large dot matrix and its manipulation. *seqdotplot* can load large sequences, take control of the size of the window on the reference genome and set the number of mismatches in it. The functions *nwalign* and *swalign* are good simulations of the well-known dynamic alignment algorithms N.W. and S.W., respectively. Whatever is the aim, the main program splits the dot matrix into smaller windows and windows that are directly connected with the main diagonal of the plot are examined. All our results are conducted with this setting ignoring windows of second priority. The majority of the diagonals, a fraction ~50%, in a sub-matrix, or window, are scanned for “1” (dot) values and the summation is kept for each one. When the sum is higher than a threshold, this window is recorded as positive. Finally, a matrix keeps the positive windows to index the regions of similarity. The threshold for the summation is analogous to the size of the window. Due to the limited performance and memory allocation of our program, reasonable sizes of sequences were tested.

## Supplementary information

Supplementary Info

## Data Availability

All data generated or analyzed during this study are included in this published article (and its Supplementary Information files).
