# Supplementary material for: Α Quantum Pattern Recognition Method for Improving Pairwise Sequence Alignment
Source: Sci Rep. 2019 May 10;9:7226. doi: 10.1038/s41598-019-43697-3 (PMC6510764; doi:10.1038/s41598-019-43697-3)
Supplement: Supplementary file 1 — Supplementary Info [file 41598_2019_43697_MOESM1_ESM.docx]

***Supplementary Material***

Manuscript Title: Α Quantum Pattern Recognition Method for Improving Pairwise Sequence Alignment

Authors: Konstantinos Prousalis & Nikos Konofaos

Affiliation: Department of Informatics, Aristotle University of Thessaloniki, Thessaloniki, Greece.

**Quantum Fourier Transform**

This is the sub-circuit of the main circuit of the QPR algorithm in Fig. S1. It is the well-known linear transformation that affects the amplitudes and phases of the base states of a qubit-system. The *R_u_* unitary transformation is defined as follows:

| $R_{u}\equiv\left[ \begin{matrix} 1 & 0 \\ 0 & e^{2\pi i/2^{u}} \end{matrix} \right]$ |  |
| --- | --- |

and its operation modifies the phase *φ =* 2*π*/2*^u^* (where $u\mathbb{\in Z}$) of a hypothetical quantum state $\left| \left. q \right\rangle\right.=\alpha\left| \left. 0 \right\rangle+ \right.\beta e^{-i\varphi}\left| \left. 1 \right\rangle\right.$ leaving unchanged the probability of measuring the orthonormal basis states of the quantum system.

Supplementary Figure S1 | The circuit model of the quantum Fourier transform.


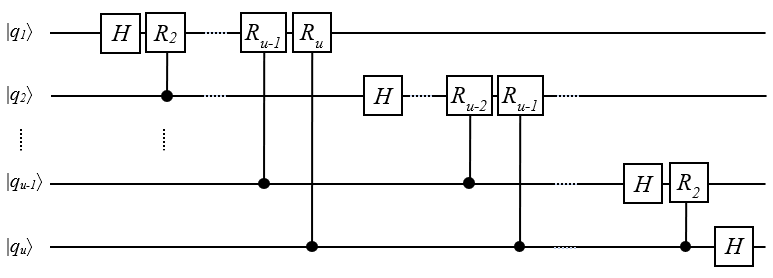


**Pseudocode of the main program**

Supplementary Table S1 Pseudocode of QPR protocol and its subroutine routineQPR.

|  | Main program |  | Quantum pseudocode of *routineQPR*(*Ω*) |
| --- | --- | --- | --- |
| *01*  *02*  *03*  *04*  *05*  *06*  *07*  *08*  *09*  *10*  *11*  *12* | Input sequences *S_R_* and *S_Q_*  Set detection parameters (*pattern_type*, *L_0_*, *Ω*)  Divide *S_R_* into *w* segments  Do {  set *window*[*i*]  run *routineQPR(Ω)*  run *routineLaue(k_1_,..,k_Ω_)*  save record(*id*, *L_D_*, x[*start,end*],y[*start,end*])  refine *BB*  *i* + +  } while (*i* < *w*)  Refine results with DP | *01*  *02*  *03*  *04*  *05*  *06* | Initialize *n*-qubit *regX*, *m*-qubit *regY* and 1-qubit *regF* registers:  $regX: \left\vert x \right\rangle$←0, *regY*: $\left\vert y \right\rangle$←0 and *regF*: $\left\vert f \right\rangle$←0  Bring superposition by Hadamard gates to *regX* and *regY*:  $H^{(n)}\left\vert0^{(n)} \right\rangle$ and $H^{(m)}\left\vert0^{(m)} \right\rangle$  Apply *BB* to *regX* and *regY*: $BB\left\vert x,y \right\rangle$  Apply measurement to *regf.*  Apply QFT: $QFT\left\vert x,y \right\rangle$  Apply measurement to *regX* and *regY*. |

**Overview of the most common dot-plot patterns**

The diversity of biological sequences can form a variety of patterns when viewed in a dot matrix plane. A schematic overview of the most common patterns is given in Fig. 3. (*a-f*) cases concern comparison of the one sequence by itself (*S_Q_*=*S_R_*) while (*g-h*) cases concern comparison of different sequences (*S_Q_*≠*S_R_*).

Supplementary Figure S2 | An overview of the most common patterns appearing in dot-plots. The first six patterns (a-f) are self-similarity dot plots and the rest (g-h) concern different sequences.


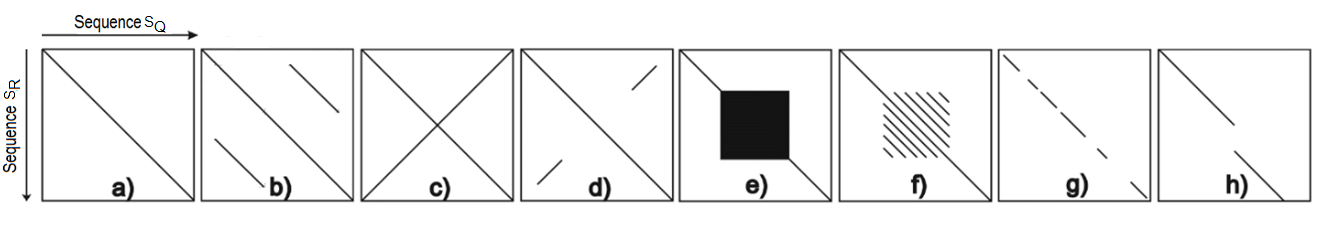


The first type-pattern (a) demonstrates a perfect similarity, (b) repeated regions in the same reading direction (a.k.a. duplications), (c) palindromic areas (e.g. ¨EVITITEPEREPETITIVE¨), (d) a partially palindromic sequence, (e) repetition of the same symbol in both sequences (a.k.a. microsatellite repeats) and (f) tandem repeats of a larger motif in both sequences (a.k.a. minisatellite patterns). (g) and (h) patterns demonstrate partial deletion or insertion (a.k.a. *indels*) in sequences due to mutations or other physical reasons.

**Common SA Strategies**

Apart from the dot-matrix method, the most prevalent SA strategies to date are the seed-and-extend and the *q*-gram filter. A brief description of these methods follows:

(a) **Dot-matrix plot:** This approach is conceptually simple in construction and the method[^16^](#ref12) itself is context-independent. Two sequences are placed vertically on the two axis of a matrix plot and each cell is marked with a dot only if the coordinates of this cell have the same letter (or token). The construction of the matrix for long sequences is time consuming as it is proportional to the product *L_Q_L_R_*. The memory allocation has 4*αL_R_* + 2*L_T_* plus 1 byte per dot, where *α* the diversity of sequence’s alphabet. Moreover, compressing techniques can reduce memory allocation significantly. Small values for *α* intensify background noise rendering hard the identification of similar regions, but filter adaptation can alleviate the problem.

(b) **Seed-and-extend:** This method makes use of shorter portions of the read, known as *seeds*, in order to find exact or inexact similarities among the sequences. The whole process can be divided into four stages: seed generation, seed mapping (seeding), extension of each matched seed, and alignment of the read to the reference sequence. The seeds are extracted from the read using different methods. Then an index structure facilitates the matching of the seeds to the reference sequence. Finally, each matching seed is further extended on both directions in order to reach neighboring matches with SW or NW algorithms. Additional criteria such as maximum mismatches or length of indels enable constrains. A scoring system usually deems the relevance among the seeds. Some prominent variations of this strategy are: (1) the *k*-mer exact match seed (BLAST and GNUMap), (2) the *k*-mer inexactly match seed (SOAP, Bowtie, BWA), (3) the *k*-mer spaced seed (RMAP, Maq), (4) the maximum extend match (MEM) seed (BWA-MEM, CUSHAW2) and (5) the adaptive seed (LAST, AMAS).

(c) ***q*-gram filter:** In this strategy multiple seeds, or *q*-grams, are used to find the best fit between the query and the reference sequence. When there is a highly mapped region by a predefined majority of *q*-grams, then only these regions are selected to get aligned by D.P. The alignment process finally will determine which matching is accepted or not, working as a filter. Thus, the extension stage is skipped. This strategy generally contains four steps: the generation of q-grams, their multiple mapping to the reference sequence, the selection of the most highly mapped regions and the filtering during the alignment. Some popular aligners with improved sensitivity are the SHRiMP and Hobbes.

**Basic ingredient algorithms**

Searching efficiently a query sequence in a database is of primary importance. Smart index tools to effectively organize the reference and/or the query sequences are necessary. Most aligners are mainly based on two index tools: hash tables and prefix/suffix trees. These indexing structures usually affect substantially the overall performance since the seeding procedure is crucially dependent on them. However, a variety of backstage auxiliary algorithms has contributed to make competitive hash map and prefix tree structures.

*(1) Hash map.* Hashing the DNA reference genome, the amount of memory required based on the number of bases in the genome *L_R_* and the mer-size of the hash *k* can be computed by 4 * (4*k* + *L_R_*). Thus, the space complexity is linearly increased with the size of the genome. For large sequences the time has a constant complexity except for collision problems in the internal structure of the hash table.

*(2) Prefix/Suffix trie*. A prefix trie, or simply a trie, is a data structure that stores all the suffixes of a string, enabling fast string matching. Certain representation of this structure, such as suffix tree (compressed version), enhanced suffix array and *FM*-index, are used to find exact matches.

*(3) Burrows-Wheeler Transform with FM-index.* The Burrows-Wheeler transform (*BWT*) combined with the FM-index can offer a great advantage in space and time. This tool operates not only as an indexing structure but also as a compression tool. *FM*-index is a compressed representation of the BWT(*s*) where *s* an arbitrary long string. *FM*-index is based on BWT and is possible to be linked with a prefix trie which provides reverse strings, or even a suffix tree, being independent of methods of inexact matches. The creation of an array of for *S_N_* strings takes *O*(*S_N_*) time and space and its sorting time with a standard method (e.g. quick-sort or merge-sort) takes *O*(*S_N_* log*S_N_*) time. Consequently, the overall time complexity for BWT is *O* (*S_N_* + (*S_N_* log *S_N_*)) for a fixed alphabet. By using backward search with BWT, it is possible to effectively mimic the top-down traversal on the prefix trie of the genome with relatively small memory footprint and to count the number of exact hits of a target string of length *L_T_* in O(*L_T_*) time independent of the size of the genome.

*(4) FMD-index.* In contrast to the bidirectional *BWT* that makes usage of two *FM*-indices, the *FMD*-index develops structures for the sequences that allow both forward and reverse indexing. The computational complexity is the same for both methods.

*(5) Directed Acyclic Word Graph.* A directed acyclic word graph (*DAWG*) for a text *T* is a minimal automaton that accepts all substrings of *T*, so it represents a complete index of the text. The construction time of this implementation is linear with respect to the size of the text and a search for a specific pattern is done in a linear time with respect to the size of the pattern. Building a prefix *DAWG* for the query sequences potentially helps to avoid repeatedly aligning identical substrings in the query, and thus improves the theoretical time complexity.

**Alignment methods**

Software alignment tools of all the categories are selected. Eleven prominent short-read aligners have been studied and are briefly presented in respect to the three most common strategies. The most significant features of each aligner are described:

**1. MAQ.** This method saves time in seed generation and mapping. The reference genome is initially scanned for exact seeding and if it fails MAQ generates spaced seeds to scan again. Each individual alignment adopts a phred-scaled quality score which is used to measure the probability of the validity of each alignment. The larger the quality score the better the alignment. The score is based on the mismatched bases over the whole length of the read.

**2. GNUMap.** Initially, the extracted reads are represented by the position weight matrix (PWM) model which is the most popular way to predict biologically meaningful sequence regions. Then, a likelihood score is assigned to any residue of any position on any given read. A quality filter will remove reads according to Solexa pipeline. A hash table is created for the genome instead of the reads which enables for the computation of a probabilistic scoring scheme. *k*-size substrings are selected in the genome to run the hash function and find matching positions in the reference genome. The matching region is confirmed by using a probabilistic version of NW algorithm. The read is scored against all possible matches in the genome and a proportional share is added to all the matching genomic locations. A posterior probability for each read is computed taking into consideration the number of occurrences of each one in the genome.

**3. SOAP/SOAP2.** The SOAP family aligners split a read into fragments, based on the number of mismatches allowed (default five), to implement the strategy using inexact match seeds. The SOAPv2 improves SOAP in speed and memory by using an index based on the BWT. The matches are located by a hash table which accelerates the searching of the BWT reference index.

**4. Bowtie.** Bowtie employs a Burrows-Wheeler index based on the full-text minute-space (FM) index which allows for small memory footprints. More specific, there is the ¨forward¨ index, which contains the BWT of a reference sequence, and the ¨mirror¨ index, which is composed of the BWT of the reversed reference sequence. This scheme is known as ¨double indexing¨ technology and its main contribution is that helps prevent excessive backtracking. Using double indexes, exactly matched seeds can be quickly identified. The algorithm consists of three phases that alternate between using the forward and mirror indices in order to find matches and extend partial alignments into full alignments. A quality score determines whether extension of a read continues. If the alignment has a quality score larger than the cutoff (default 70), the extension on the match seed is stopped. This quality score constraint removes a lot of matched seeds for continuous extension alignment as early as possible. This quality-aware backtracking algorithm allows mismatches and favors high-quality alignments.

**5. BWA.** BWA generates *k*-mer inexactly match seeds with a default of two mismatches allowed in each seed. The adopted index structure is the prefix DAWG which efficiently accomplishes the mapping. Seed extension for highly repetitive sequence regimes is skipped. In brief, extensions are abandoned when the length of the overlapped region is shorter than the length of any previous successfully aligned regions in the reference genome.

**6. SHRiMP.** This tool allows multiple matching seeds, known as q-grams, in order to start the alignment process incorporating spaced seeds during q-gram’s generation. This technique improves mapping sensitivity. SHRiMP regards candidates for each read by top ranking the q-grams using their numbers of hits on the reference genome. A SW based algorithm accomplishes the alignment process.

**7. CUSHAW2.** MEM seeds are detected from the FM-index of a read or a reference sequence. The super-maximal exact matches (SMEMs) are strings that are not included in any other MEMs of the read. They are selected for seed mapping and extension. Invalid extensions of the other MEMs are prevented by using SMEMs. If SMEMs don’t give an alignment, a new round of seed’s generation is conducted. The extension process may be interrupted if the difference between the best alignment score in the extension and the score at that point is larger than a predefined value. If the extension ends up with a large and acceptable score, the alignment is considered a successful one. A filtering mechanism excludes very short seeds not satisfying a minimum length to avoid invalid extensions.

**8. AMAS.** The adopted seeds are non-overlapping adaptive seeds which are generated one by one through scanning the read. When the mapping frequency of the seed is less than a predefined frequency threshold, the seed is selected as an adaptive seed and the next new seed is initiated. The last seed of the read is usually shorter and gives many more matches but it is usually excluded due to the predefined threshold (cutoff).

**Simulation**

The proposed simulator is available in the GitHub repository. The source code is written in Matlab and is uploaded in the following link: <https://github.com/konprou/PRonDotPlot>

A graphical user interface makes much easier the pattern recognition parameterization of the query sequences and provides a visual perception of detection’s sensitivity for pairwise alignment.

In the following screenshots, Figure 3, two comparison examples are depicted between the YASS tool and our own tool to display or demonstrate the possibilities of our simulator on regions with highly distorted diagonals. YASS is a genomic similarity search tool, for nucleic (DNA/RNA) sequences in fasta or plain text format and produces local pairwise alignments. Like most of the heuristic pairwise local alignment tools for DNA sequences (FASTA, BLAST, PATTERNHUNTER, BLASTZ/LASTZ, LAST ...), YASS uses seeds to detect potential similarity regions, and then tries to extend them to local alignments.

Supplementary Figure S3 | Screen shots comparing YASS to our own tool.

| 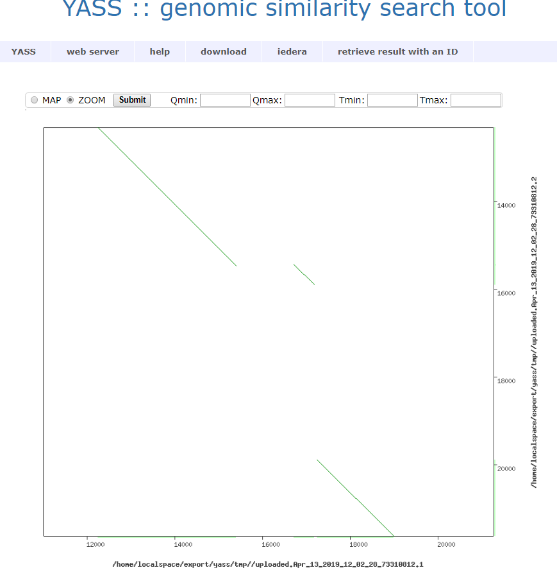 | 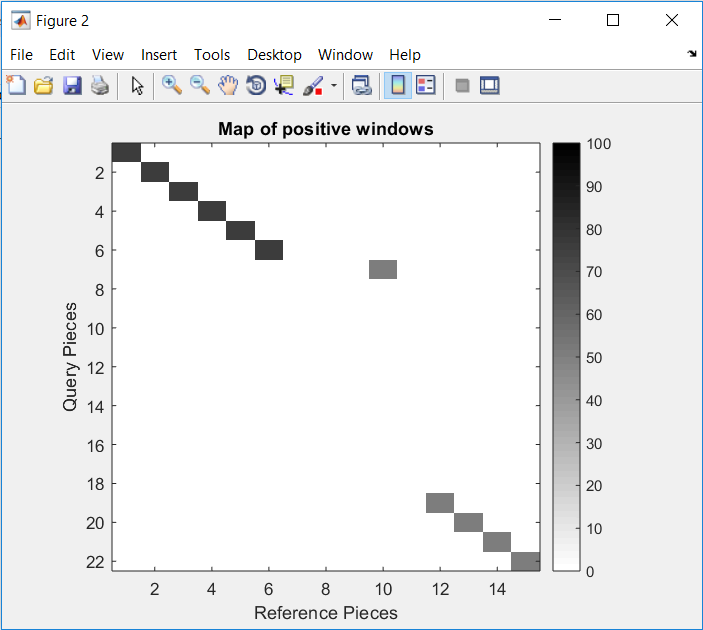 |
| --- | --- |
| *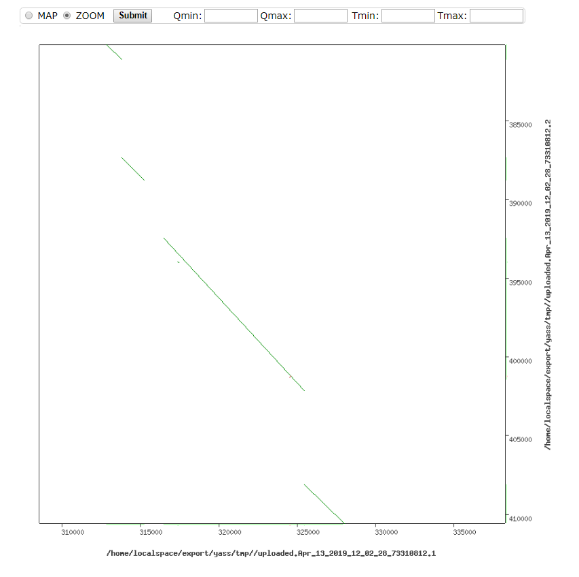* | *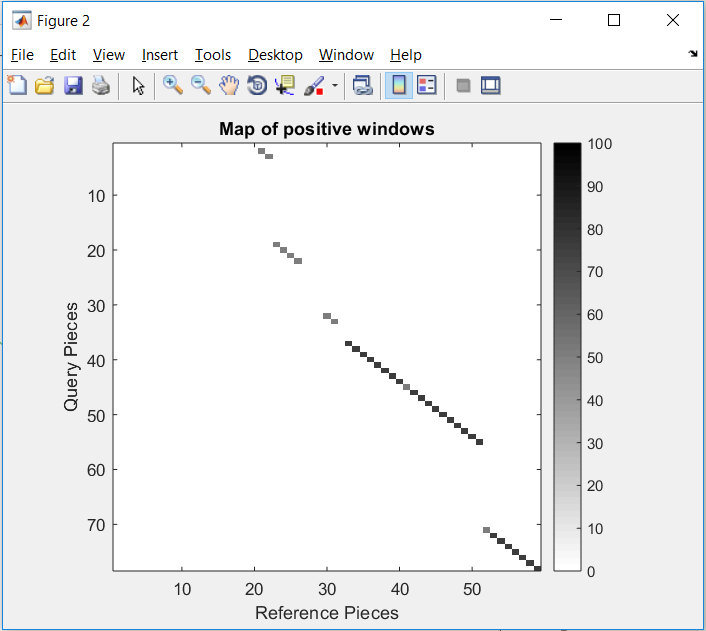* |

In the matlab dotplot, each positive window is depicted as a shade of white/black (see color bar) which describes the percentage of the length of the highest detected diagonal within the window. A window of the size 200 corresponds to a pairwise alignment between strings of 200 symbols. To sum up, our simulator has the following control characteristics:

1. Controls the size of the searching window.
2. Controls the coverage of the window in terms of how many diagonals to searched.
3. Controls the area of the genome to be searched.
4. Controls the examination of the nearby windows along the main diagonal if requested.
5. Provides a general graphical view in terms of windows detected as positive.
